# Efficacy of PSMA ligand PET-based radiotherapy for recurrent prostate cancer after radical prostatectomy and salvage radiotherapy

**DOI:** 10.1186/s12885-020-06883-5

**Published:** 2020-04-29

**Authors:** Ann-Kathrin Oehus, Stephanie G. C. Kroeze, Nina-Sophie Schmidt-Hegemann, Marco M. E. Vogel, Simon Kirste, Jessica Becker, Irene A. Burger, Thorsten Derlin, Peter Bartenstein, Matthias Eiber, Michael Mix, Christian la Fougère, Claus Belka, Stephanie E. Combs, Anca-Ligia Grosu, Arndt-Christian Müller, Matthias Guckenberger, Hans Christiansen, Christoph Henkenberens

**Affiliations:** 1 Department of Radiotherapy and Special Oncology, Hannover Medical School, Carl-Neuberg-Str. 1, 30629 Hannover, Germany; 2 Department of Radiation Oncology, University Hospital Zürich, University of Zurich, Zurich, Switzerland; 3 Department of Radiation Oncology, University Hospital LMU Munich, Munich, Germany; 4 Department of Radiation Oncology, Technical University Munich, Munich, Germany; 5 Department of Radiation Oncology, University of Freiburg, Freiburg, Germany; 6 German Cancer Consortium (DKTK) Partner Site Freiburg, German Cancer Research Center (DKFZ), Heidelberg, Germany; 7 Department of Radiation Oncology, University Hospital Tübingen, Tübingen, Germany; 8 Department of Nuclear Medicine, University Hospital Zürich, Zürich, Switzerland; 9 Department of Nuclear Medicine, Hannover Medical School, Hanover, Germany; 10 Department of Nuclear Medicine, University Hospital LMU Munich, München, Germany; 11 Department of Nuclear Medicine, Technical University Munich, München, Germany; 12 Department of Nuclear Medicine, University of Freiburg, Freiburg, Germany; 13 Department of Nuclear Medicine, University Hospital Tübingen, Tübingen, Germany; 14 Cluster of Excellence iFIT (EXC 2180) “Image Guided and Functionally Instructed Tumor Therapies”, University of Tübingen, Tübingen, Germany; 15 German Cancer Consortium (DKTK) Partner Site Tübingen, Tübingen, Germany; 16 German Cancer Consortium (DKTK) Partner Site Munich, German Cancer Research Center (DKFZ), Heidelberg, Germany; 17 Institute of Innovative Radiotherapy (iRT), Oberschleissheim, Germany

**Keywords:** PSMA, Radiotherapy, Prostate cancer, Oligometastases, Recurrence, Radical prostatectomy

## Abstract

**Background:**

A substantial number of patients will develop further biochemical progression after radical prostatectomy (RP) and salvage radiotherapy (sRT). Recently published data using prostate-specific membrane antigen ligand positron emission tomography (PSMA - PET) for re-staging suggest that those recurrences are often located outside the prostate fossa and most of the patients have a limited number of metastases, making them amenable to metastasis-directed treatment (MDT).

**Methods:**

We analyzed 78 patients with biochemical progression after RP and sRT from a retrospective European multicenter database and assessed the biochemical recurrence-free survival (bRFS; PSA < nadir + 0.2 ng/ml or no PSA decline) as well as the androgen deprivation therapy- free survival (ADT-FS) using Kaplan-Meier curves. Log-rank test and multivariate analysis was performed to determine influencing factors.

**Results:**

A total of 185 PSMA – PET positive metastases were detected and all lesions were treated with radiotherapy (RT). Concurrent ADT was prescribed in 16.7% (13/78) of patients. The median PSA level before RT was 1.90 ng/mL (range, 0.1–22.1) and decreased statistically significantly to a median PSA nadir level of 0.26 ng/mL (range, 0.0–12.25; *p* < 0.001). The median PSA level of 0.88 ng/mL (range, 0.0–25.8) at the last follow-up was also statistically significantly lower (*p* = 0.008) than the median PSA level of 1.9 ng/mL (range, 0.1–22.1) before RT. The median bRFS was 17.0 months (95% CI, 14.2–19.8). After 12 months, 55.3% of patients were free of biochemical progression. Multivariate analyses showed that concurrent ADT was the most important independent factor for bRFS (*p* = 0.01). The median ADT-FS was not reached and exploratory statistical analyses estimated a median ADT-FS of 34.0 months (95% CI, 16.3–51.7). Multivariate analyses revealed no significant parameters for ADT-FS.

**Conclusions:**

RT as MDT based on PSMA - PET of all metastases of recurrent prostate cancer after RP and sRT represents a viable treatment option for well-informed and well-selected patients.

## Background

Salvage radiotherapy (sRT) represents the standard of care for patients with a biochemical relapse after radical prostatectomy (RP) of localized prostate cancer (PCa) [1]. Nevertheless, a substantial number of patients will not benefit permanently from sRT and will develop biochemical progression [2, 3]. Recently published data suggest that early recurrences are often located outside the prostate fossa [4–6], and a large proportion of these patients (40–70%) have a limited number of metastases, making them amenable to metastasis-directed treatment (MDT) [7]. These cases are usually considered oligorecurrent disease. Despite the lack of a biologically defined oligometastatic status and a strict clinical definition, the evidence for MDT for patients with a generally accepted imaging-based cut-off of five metastases - outside large randomized prospective trials - is consistently increasing [8, 9]. Recent data showed a positive effect on the clinical outcome for MDT with low toxicity, although staging with positron emission tomography (PET) with prostate-specific membrane antigen (PSMA) radio ligands was not available and the number of metastases could therefore be underestimated [10, 11].

Furthermore, the successful implementation of PSMA -PET – outperforming all other imaging modalities [12] – significantly improves patient selection for MDT and subsequently leads to high acceptance of MDT in patients with limited prostate cancer recurrence [13]. PSMA - PET allows individualizing treatment concepts that aim to improve PSA progression-free survival, defer the initiation of androgen deprivation therapy ADT and potentially cure the patient [14]. In addition, the optimal timing of initiation of ADT for asymptomatic biochemically progressive disease after RP and sRT remains unknown [1]. Importantly, it has been shown that ADT could be safely deferred in a relevant proportion of patients by MDT, which may also improve quality of life (QoL) [8, 9].

However, the potential usefulness of a PSMA - PET-guided MDT approach in the clinical setting even after sRT (i.e., in patients with a longer treatment history than in previously investigated approaches) is underexplored. Therefore, we investigated the efficacy and safety of definitive radiotherapy (RT) for PSMA - PET-detected oligometastatic disease after both RP and prior sRT in a retrospective European multicenter study.

## Methods

This retrospective multicenter study was approved by the institutional review boards of all participating centers (BASEC-Nr. 2017–01499). Included patients (*n* = 379) were treated with definitive PSMA - PET-based RT as MDT between 04/2013 and 01/2018 in 6 academic centers in Switzerland and Germany. In the present analysis, we included 78 patients with biochemical progression after initial RP plus sRT and subsequent diagnosis of oligorecurrent PCa on the basis of PSMA - PET. All patients presented with no evidence of distant metastases (M0) at initial diagnosis and salvange radiotherapy of the prostatic bed. PCa recurrence was defined as nodal or extranodal metastases (N1 or M1a/1b/1c) in PSMA - PET. PET-CT or PET-MRI was performed with ^68^Ga radiolabelled PSMA- ligand. Any PSA level at the time of RT was accepted. Oligorecurrent disease was defined as ≤5 visceral or bone metastases; there was no limit on lymph node metastases. All cases were discussed and approved for RT by the local multidisciplinary uro-oncologic boards. The patients’ characteristics are summarized in Table 1.

**Table 1:** Patient characteristics

Age at PCa diagnosis	Median (range)
	64 (48–78)
Initial T-stage	n (%)
T2a/b	6 (7.7)
T2c	22 (28.2)
T3a	18 (23.1)
T3b	32 (41.0)
Initial N stage	n (%)
N0	62 (79,5)
N1	16 (20,5)
Surgical margins	n (%)
R0	48 (61,5)
R1	30 (38,5)
Initial risk group	n (%)
Intermediate	3 (3.9)
High	29 (37.2)
Very high	46 (58.8)
Initial PSA (ng/ml)	Median (range)
	11.4 (2.8–231.0)
First PSA after RP (ng/ml)	Median (range)
	0.07 (0.0–1.9)
Interval in months from RP to sRT	Median (range)
	11.16 (3.1–172.6)
PSA nadir after sRT (ng/ml)	Median (range)
	0.84 (0.0–12.2)
Interval in months from sRT to PSA recurrence	Median (range)
	22.7 (3.0–136.6)
Biochemical non-response after sRT	n (%)
	4 (5.1)
PSA level at PSMA-PET imaging (ng/ml)	Median (range)
	1.9 (0.1–22.1)
Patients with ADT at PSMA-ligand PET imaging	n (%)
	3 (3.9)
PSA-dt at time of PSMA-PET imaging (months)	n (%)
< 3 (n)	3 (3.9)
3–6 (n)	32 (41.0)
> 6–12 (n)	23 (29.5)
> 12 (n)	16 (20.5)
unknown	4 (5.1)

*ADT* androgen deprivation therapy, *dt* doubling time, *PCa* prostate cancer, *PSMA-PET* prostate-specific membrane antigen positron emission tomography, *PSA* prostate-specific antigen, *RP* radical prostatectomy, *sRT* salvage radiotherapy

### PET imaging

Each patient received PET imaging with a ^68^Ga-labeled PSMA ligand [15], and imaging was performed according to the joint EANM and SNMMI guidelines [16]. PSMA - PET scans were acquired in conjunction with either contrast-enhanced or low-dose computed tomography (PET/CT; 87.2%, 68/78) or magnetic resonance imaging (PET/MRI; 12.8%, 10/78). Visual assessment of focally increased tracer uptake higher than the surrounding background activity was used as the criterion for malignancy [8].

### Radiotherapy treatment

Patients were treated with stereotactic body radiation therapy (SBRT) or with conventionally fractionated RT (CF-RT), including either a conventionally fractionated simultaneously integrated boost (SIB) or a sequential SBRT boost to the PET-positive lesion. Definitive RT was delivered to all PSMA ligand-positive lesions. Irradiation was performed at the discretion of the participating center in terms of radiation dose, elective nodal volumes, and type and length of concurrent ADT. The prescribed radiotherapy dose was converted to EQD2 in Gy using an α/β ratio of 1.5 [17].

### Follow-up and endpoints

Biochemical recurrence-free survival (bRFS) was measured from the last day of RT to the diagnosis of biochemical recurrence and defined according to PSA failure after RP [18]: PSA ≥ 0.2 ng/ml above the PSA nadir following RT. When serum PSA did not respond to RT, the pre-RT level with an increase of ≥0.2 ng/ml was defined as bRFS. Follow-up was performed according to institutional protocols, with regular serum PSA measurements and clinical follow-up visits. Secondary outcomes were ADT-free survival (ADT-FS), overall survival (OS) and toxicity. The timing of imaging at biochemical recurrence after RT, as well as the initiation of local and/or systemic therapies, was at the discretion of the local multidisciplinary uro-oncologic board. RT-associated toxicity was analyzed using the National Cancer Institute Common Terminology Criteria for Adverse Events (CTCAE) v4.0 [19].

### Statistical analysis

For statistical analysis, SPSS Statistics v25.0 (IBM, Armonk, New York, USA) was used. We used the paired Student’s *t* test to compare pre-RT with post-RT parametric parameters and the Wilcoxon signed-rank test when data were not normally distributed. The time to event data was calculated using the Kaplan-Meier method. Established factors for treatment failure after sRT [20, 21] were analyzed with log rank test in univariate analyses, and significant factors were further assessed with multivariate analyses to identify independent variables for bRFS and ADT-FS. *P*-values of < 0.05 were considered statistically significant.

## Results

### Result of PSMA ligand PET staging and therapy of metastases

A total of 185 PSMA ligand positive metastases were detected and treated with RT: 41.6% (77/185) were pelvic lymph node metastases, 27.6% (51/185) were periaortic lymph node metastases, 24.3% (45/185) were bone metastases, and 6.5% (12/185) were visceral metastases. A total of 58.8% of patients (46/78) had only lymph node metastases, 32.1% (25/78) of patients had only bone metastases, 3.8% (3/78) of patients had lymph node and bone metastases, 2.6% (2/78) of patients had visceral metastases only, and 2.6% (2/78) of patients had visceral and lymph node metastases.

Concurrent ADT was prescribed in 16.7% (13/78) of patients and ADT was deferred in the remaining patients. Furthermore, additive chemotherapy with docetaxel was administered in 30.8% (4/13) of patients with concurrent ADT. The majority of the patients (57.7%; 45/78) received CF-RT, 20.5% (16/78) received SBRT, 12.8% (10/78) received CF-RT with SBRT boost, and 9% (7/78) of the patients were treated with CF-RT and a simultaneous integrated boost (SIB).

Table 2 summarizes the results of the PSMA ligand PET results and therapy of the metastases.

**Table 2:** PSMA-ligand PET results and radiotherapy of metastases

PSMA-ligand PET results	N (%)
Number of PSMA-ligand positive lesions	185 (100
Total number of LNs	128 (69.2)
Iliac LNs	67 (36.2)
Obturator LNs	6 (3.2)
Perirectal LNs	4 (2.2)
Periaortic/interaortocaval LNs	51 (27.6)
Total number of bone metastases	45 (24.3)
Pelvic bone	24 (13.0)
Spinal bone	10 (5.4)
Other	11 (5.9)
Number of visceral metastases	12 (6.5)
Concurrent ADT at radiotherapy	13 (16.7)
Radiotherapy of metastases	N (%)
Radiotherapy of LNs	128 (100)
CF-RT	95 (74.2)
SBRT	5 (3.9)
CF-RT plus SBRT	15 (11.7)
CF-RT with SIB	13 (10.2)
Median dose, EQD2/1.5 Gy (range)	50.9 (50.0–76.1)
	N (%)
Radiotherapy of bone metastases	45 (100%)
CF-RT	26 (57.8)
SBRT	15 (33.3)
CF-RT plus SBRT	0 (0)
CF-RT with SIB	4 (8.9)
Median dose, EQD2/1.5 Gy (range)	51.4 (46.4–108.8
	N (%)
Radiotherapy of visceral metastases	12 (100)
CF-RT	8 (66.7)
SBRT	4 (33.3)
CF-RT plus SBRT	0
CF-RT with SIB	0
Median dose, EQD2/1.5 Gy (range)	64.7 (57.8–85.0)

*ADT* androgen deprivation therapy, *CF-RT* conventionally fractionated radiotherapy type, *Gy* Gray, *LNs* lymph node metastases, *PCa* prostate cancer, *PSMA-PET* prostate-specific membrane antigen positron emission tomography, *PSA* prostate-specific antigen, *RP* radical prostatectomy, *sRT* salvage radiotherapy, *SBRT* stereotactic body radiation therapy, *SIB* simultaneous integrated boost

### Patients’ outcome

The median follow-up was 16 months (3–54). Overall survival (OS) was 97.4% after 2 years; 2 patients died of progressive metastatic PCa. The median PSA level before RT was 1.90 ng/mL (range, 0.1–22.1) and decreased statistically significantly to a median PSA nadir level of 0.26 ng/mL (range, 0.0–12.25; *p* < 0.001). The median PSA level of 0.88 ng/mL (range, 0.0–25.8) at the last follow-up was also statistically significantly lower (*p* = 0.008) than the median PSA level of 1.9 ng/mL (range, 0.1–22.1) before RT. Moreover, 20.5% (16/78) of all patients had a PSA level < 0.07 ng/mL at the last available follow-up. Nine of these 16 patients (56.3%) were ADT naïve.

**Fig. 1:**
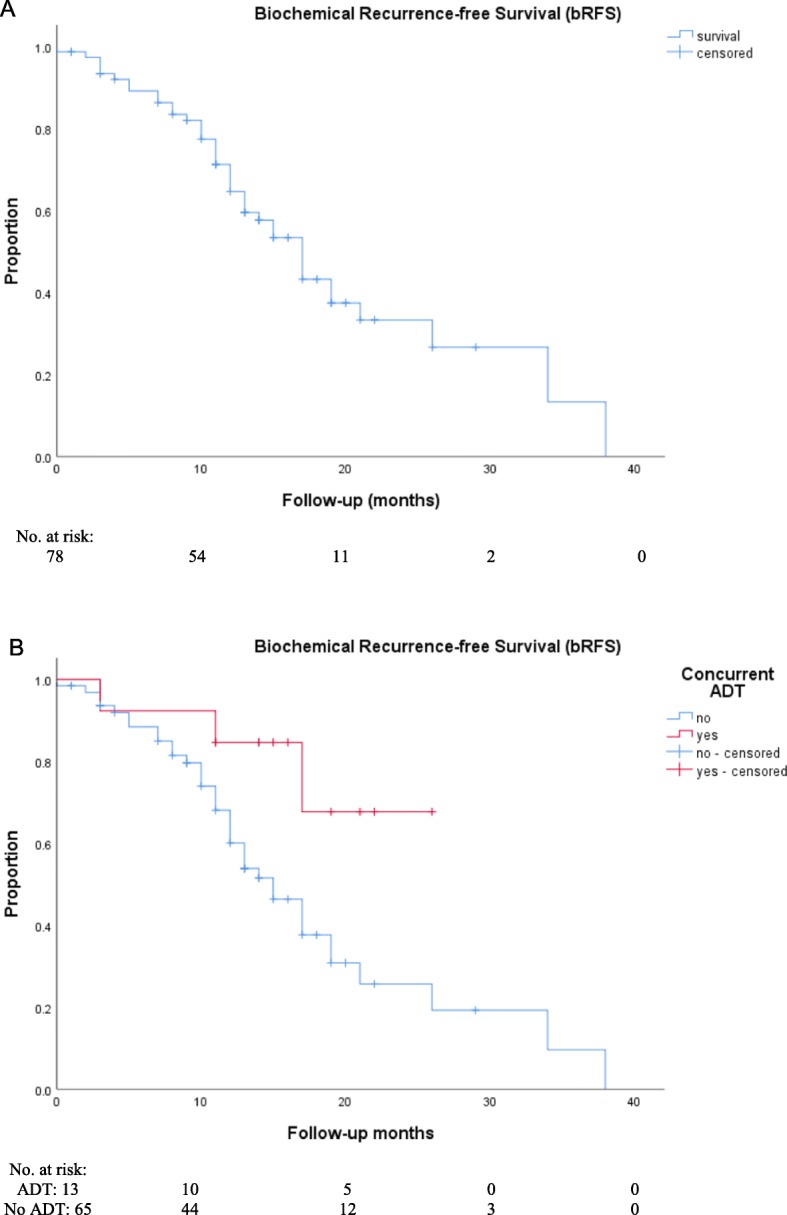
Kaplan-Meier curves of biochemical recurrence-free survival (bRFS) after 68 Ga-labeled PSMA ligand PET directed radiotherapy of prostate cancer (**a**) with or without ADT (*p* = 0.03; **b**)

A total of 57.7% (42/78) of patients were classified as having biochemically progressive disease after RT. The median PSA level at biochemical progression was 0.37 ng/mL (range, 0.1–3.6). The median bRFS was 17.0 months (95% CI, 14.2–19.8; Fig. 1). After 12 months, 55.3% of patients were free of biochemical progression. Multivariate analyses showed that concurrent ADT was the most important independent factor for bRFS (*p* = 0.01). The detailed results of the statistical analyses are shown in Table 3. Furthermore, 83.3% (35/42) of patients with biochemically progressive disease underwent restaging, which was performed with the exception of one patient (2.9%) with PSMA - PET. The staging revealed new metastases in 85.7% (30/35) of patients and in 14.3% (5/35) of patients PSMA – PET detected no metastases. The resulting treatment concepts for patients with biochemical progression were as follows: in 50% (21/42) of patients, ADT was initiated, and 14.3% (6/42) of patients started observation with regular PSA measurements, and in 9.5% (4/42) of patients, repeated RT was performed. Furthermore, 9.5% (4/42) of patients received ADT plus repeated RT; 7.1% (3/42) of patients received taxane-based chemotherapy, and 2.4% (1/42) of patients received taxane-based chemotherapy plus ADT. Additionally, in 4.8% (2/42), a therapy with radium-223 dichloride was initiated, and 2.4% (1/42) of patients received a secondary hormonal ablation with enzalutamide.

**Table 3:** Results of uni- and multivariate analyses for biochemical progression-free survival (bRFS)

	Univariate analysis	Multivariable analysis	
	*p* value	*p* value	OR, (95% CI)
Initial T stage (≤T2 vs ≥ T3)	0.02	0.50	1.43 (0.50–4.11)
Initial N stage (N0 vs N1)	0.07	0.07	3.75 (0.89–15.81)
Initial PSA level in ng/ml (≤20 vs > 20)	0.36		
PSA nadir after RP (≤0.07 ng/mL vs > 0,07 ng/mL)	0.04	0.14	2.24 (0.78–6.45)
Number of removed LN at RP (≤15 vs > 15)	0.32		
Initial Risk Group (intermediate+high risk vs. very high risk)	0.15		
PSA doubling time (≤6 months, > 6 months)	0.46		
Radiotherapy type (CF-RT vs. SBRT)	0.11		
No. of irradiated metastases (1 vs > 1)	0.37		
Type of metastases (lymph node vs bone)	0.18		
Concurrent ADT (yes vs no)	0.03	0.01	7.86 (1.51–40.79)
RT-Dose (≤50 Gy vs > 50 Gy)	0.74		

*ADT* androgen deprivation therapy, *CF-RT* conventionally fractionated radiotherapy type, *dt* doubling time, *Gy* Gray, *LN* lymph nodes, *PSA* prostate-specific antigen, *RP* radical prostatectomy, *sRT* salvage radiotherapy, *SBRT* stereotactic body radiation therapy, *SIB* simultaneous integrated boost

**Fig. 2:**
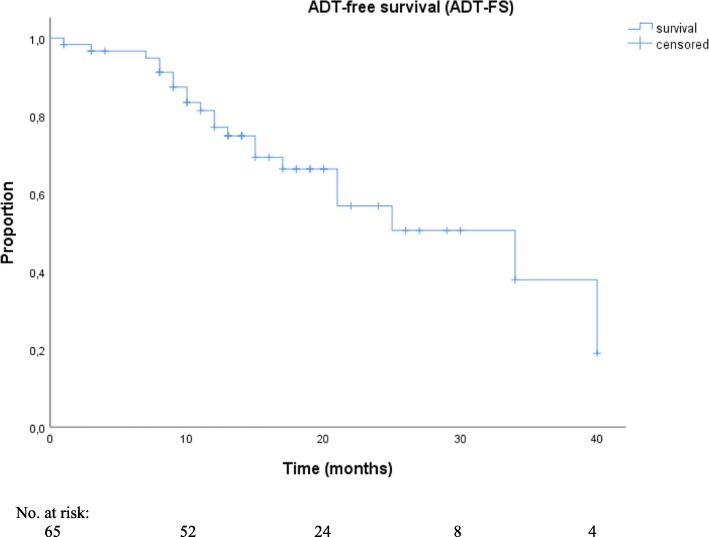
Kaplan-Meier curves of androgen deprivation therapy-free survival (ADT-FS) after ^68^Ga-labeled PSMA PET-directed radiotherapy

For ADT-FS analyses, patients among ADT at time of RT (3.9%; 3/78) or patients who received concurrent ADT +/− docetaxel (16.7%; 13/78) for RT were excluded. The median ADT-FS was not reached because less than half of the patients (38.7%; 24/62) were in need of ADT at their last follow-up visit. Exploratory statistical analyses estimated a median ADT-FS of 34.0 months (95% CI, 16.3–51.7, Fig. 2). None of the significant parameters of the univariate analyses reached significance in multivariate analyses. The detailed results of the statistical analyses are shown in Table 4.

**Table 4:** Results of uni- and multivariate analyses for androgen deprivation therapy-free survival (ADT-FS)

	Univariate analysis	Multivariable analysis	
	*p* value	*p* value	OR, (95% CI)
Initial T stage (≤T2 vs ≥ T3)	0.07		
Initial N stage (N0 vs N1)	0.95		
Initial PSA level in ng/ml (≤20 vs > 20)	0.26		
PSA nadir after RP (≤0.07 ng/mL vs > 0,07 ng/mL)	0.05	0.20	2.02 (0.68–5.99)
Number of removed LN at RP (≤15 vs > 15)	0.31		
Initial risk group (intermediate+high risk vs. very high risk)	0.02	0.10	2.58 (0.8–7.98)
PSA dt (≤6 months, > 6 months)	0.10		
Radiotherapy type (CF-RT vs., SBRT)	0.73		
No. of irradiated metastases (1 vs > 1)	0.51		
Type of metastases (Lymph node vs bone)	0.12		
RT dose (≤50 Gy vs > 50 Gy)	0.44		

*ADT* androgen deprivation therapy, *CF-RT* conventionally fractionated radiotherapy type, *dt* doubling time, *Gy* Gray, *LN* lymph nodes, *PSA* prostate-specific antigen, *RP* radical prostatectomy, *sRT* salvage radiotherapy, *SBRT* stereotactic body radiation therapy, *SIB* simultaneous integrated boost

### Toxicity

Acute grade III toxicity was not observed; 1.3% (1/78) of patients developed acute genitourinary toxicity grade II. Acute gastrointestinal toxicity grade II occurred in 1.3% (1/78) of patients, and acute gastrointestinal toxicity grade I occurred in 1.3% (1/78) of patients. Late grade III gastrointestinal toxicity occurred in 1.3% (1/78) of patients, and grade II genitourinary toxicity in 1.3% (1/78) of patients.

## Discussion

The implementation of PSMA ligand imaging has substantially improved the diagnostic accuracy for the detection of (oligo) metastatic PCa at low PSA levels [5, 22], leading to the recent guidelines by the European Organization for Research and Treatment of Cancer (EORTC) demanding modern imaging methods for trials investigating MDT in oligometastatic PCa [23]. Although large randomized prospective studies are lacking, approximately two-thirds of the experts at the 2017 Advanced Prostate Cancer Consensus Conference considered MDT as a treatment option for patients with oligorecurrent PCa [13].

A substantial number of patients will develop further biochemical progression after sRT [2, 3], and controversy still exists about the optimal timing of initiation of palliative ADT regarding asymptomatic metastatic patients because of the lack of prospective trials from the PSA era [1]. Furthermore, ADT alone offers no curative potential [1] and significantly impairs QoL in a relevant number of patients [24]. Therefore, MDT to all detectable lesions might shift the treatment concept from palliative to potentially curable [10]. Smaller prospective trials with heterogeneous patient collectives, one with choline PET imaging [10], one with PSMA ligand PET imaging [25] and one with sodium fluoride (NA-F) PET imaging [11], showed encouraging results for MDT for oligometastatic prostate cancer.

To the authors’ best knowledge, the assessed subset from a large retrospective multicenter database including only patients with oligometastatic disease after RP and sRT treated with PSMA ligand guided RT is the first analysis that showed a significant improvement of the PSA levels. The median PSA levels at the last follow-up visit were significantly lower than the PSA levels prior to RT (1.90 vs. 0.88; *p* = 0.008). Furthermore, a significant number of patients receiving RT alone could be spared ADT treatment for an estimated median time of 34 months. Our results are better than the results of the STOMP trial, which showed a median ADT-FS of 13.0 months. However, for the STOMP trial, the median PSA was 5.3 ng/ml, and PSMA ligand imaging was not available, thereby decreasing the likelihood of identifying a truly oligometastatic cohort of patients [10]. The POPSTAR trial reported a 2-year ADT-FS of 48%, which is slightly worse than the results we observed, possibly due to a higher proportion of patients with bone metastases. Furthermore, imaging was performed with Na-F PET. Na-F PET imaging outperforms conventional imaging for bone metastases but cannot depict nodal metastases, increasing the likelihood of including patients with bone metastases but underestimating lymph node metastases, possibly leading to a patient cohort with unfavorable prognosis [11].

The administration of concurrent ADT in this situation is currently unknown. RT with concurrent ADT might improve bRFS and OS for patients with low volume disease according to the CHAARTED criteria as extrapolated from the Stampede trial [26], although the study only included patients treated with slightly hypofractionated RT of the prostate. Furthermore, the study protocol provided no local therapies to asymptomatic metastases. On the other hand, the STOMP [10] and POPSTAR trials [11], as well as the data published by Kneebone et al. [25], demonstrated that MDT alone might delay ADT for a relevant period. However, patients with MDT alone develop biochemical progression earlier than patients with MDT plus ADT. The impact on OS of MDT alone remains unknown due to the short follow-up and small sample sizes in the few prospective trials [10, 11, 25]. There might be concern that patients who do not respond well to MDT might develop widespread metastases with unfavorable prognoses. On the other hand, approximately half of the patients will develop oligoprogressive disease after MDT [10], making these patients amenable to repeated MDT [27]. Furthermore, the presented cohort had PSMA ligand imaging for staging purposes, and fewer metastases should be missed compared to conventional imaging and choline PET [4, 5, 22], indicating well-selected patients. Moreover, no data about the biological evolution of prostate cancer are available in the context of prior local therapies such as RP and sRT. Exploratory analyses for patients with metachronous metastases after initial curative local therapy revealed no OS benefit for escalated systemic therapy using either the combination of ADT + docetaxel [28, 29] or ADT + enzalutamide [30] compared to ADT alone, indicating a different biology.

We did not find a statistically significant predictor for ADT-FS in multivariate analyses, such as location (bone vs. lymph node) or number of metastases. Neither Ost et al. [10] nor Kneebone et al. [25] identified any clinical parameter significantly associated with prolonged ADT-FS, likely because the number of enrolled patients (62 and 57, respectively) was too small for sufficient statistical subgroup analyses. Nevertheless, a recently published SEER database analysis suggested that patients with M1a tumor stage have a significantly greater clinical benefit from local therapies to the prostate than patients with M1b tumor stage [31]. Additionally some patients presented with progression among ADT at PSMA ligand staging and must be considered as early castration-resistant. Data about RT as MDT on this oncological situation are rare, although Berghen et al. recently yielded first information that RT substantially postponed next-line systemic treatment [32]. In general the definition of oligometastases for prostate cancer is controversial and there is no general agreement between different experts panels [13, 33]. The hypothesis generating phase II STOMP trial included patients up to three nodal or bone metastases based upon Choline PET staging [10], whereas the expert panel of the APCCC 2017 did not reach consensus regarding a numerical definition of oligometastasis, and 61% of the panelists voted for a limited number of bone and/or lymph nodes metastases that influences treatment decisions [13]. The expert panel of the Italian Association of Radiotherapy and Clincial Oncology (AIRO) reached a consensus of 100% for metachronous oligometastatic castration-sensitive prostate cancer with primary tumor controlled and up to three metastases (node or bone) [33]. Particularly in comparison to the latter results of the AIRO expert panel, we used a broader definition of oligometastases and included patients with a more advanced cancer burden. Furthermore, we there was no limit on lymph node metastases. Therefore, we cannot rule out that some patients had little benefit due to our definition of oligometastatic PCa. On the other hand the observed clinical outcomes are promising and prospective trials using PSMA ligand staging will have to investigate that.

The retrospective nature has inherent limitations and might have introduced selection bias. Furthermore, the median follow-up of 16 months and the sample size of 78 patients limited the statistical power, although the observed clinical results are robust and contribute significantly to the discussion of PSMA ligand guided MDT after RP and sRT in a quickly changing clinical field. In addition, the study included a selected cohort with mainly high-risk patients. Therefore, caution should be taken when generalizing the observed results for patients with intermediate- or low-risk PCa. Because we used clinical real-life data from an observational study, the RT treatment was heterogeneous regarding radiotherapy dose and field size, as well as the use of concurrent ADT. Prospective trials investigating the addition of ADT, the size of RT fields and the radiotherapy dose are warranted objectives in the field of relapsed PCa after RP and sRT at this time.

## Conclusion

Even in a clinical setting after both RP and prior sRT, PSMA - PET-based RT for recurrent PCa with limited tumor burden was effective and safe. RT alone delayed the initiation of ADT longer than in other cohorts. RT of all lesions after RP and sRT based on PSMA - PET represents a viable treatment option for well-informed and well-selected patients, including a close follow-up schedule, particularly after RT alone.

## Data Availability

Please contact the corresponding author for study data, which will be granted upon reasonable request.

## Abbreviations

ADT: Androgen deprivation therapy
ADT-FS: Androgen deprivation therapy- free survival
bRFS: Biochemical recurrence-free survival
CF-RT: Conventionally fractionated RT
CTCAE: Common Terminology Criteria for Adverse Events
Dt: Doubling time
EORTC: European Organization for Research and Treatment of Cancer
Gy: Gray
LNs: Lymph node metastases
MDT: Metastasis-directed treatment
NA-F: Sodium fluoride
OS: Overall survival
PSA: Prostate-specific antigen
PCa: Prostate cancer
PET: Positron emission tomography
PSMA: Prostate-specific membrane antigen
PSMA PET: Prostate-specific membrane antigen ligand positron emission tomography
QoL: Quality of life
RP: Radical prostatectomy
RT: Radiotherapy
SBRT: Stereotactic body radiation therapy
SIB: Simultaneously integrated boost
sRT: Salvage radiotherapy
